# Enteric Viruses in Ready-to-Eat Packaged Leafy Greens

**DOI:** 10.3201/eid1611.100877

**Published:** 2010-11

**Authors:** Kirsten Mattison, Jennifer Harlow, Vanessa Morton, Angela Cook, Frank Pollari, Sabah Bidawid, Jeffrey M. Farber

**Affiliations:** Author affiliations: Health Canada, Ottawa, Ontario, Canada (K. Mattison, J. Harlow, V. Morton, S. Bidawid, J.M. Farber);; University of Ottawa, Ottawa (K. Mattison, V. Morton, J.M. Farber);; Public Health Agency of Canada, Guelph, Ontario, Canada (A. Cook, F. Pollari)

**Keywords:** Norovirus, viruses, rotavirus, lettuce, leafy greens, enteric infections, Canada, letter

**To the Editor:** Fresh produce increasingly has been implicated in viral disease outbreaks (*1*). In some instances, lettuce was contaminated before wholesale distribution (*1*). Enteric viruses can be introduced in the field if produce is exposed to human waste. Processed and packaged produce can be contaminated if equipment or wash water is not effectively sanitized. Fewer than 10 infectious viral particles are sufficient to cause disease (*2*), and these organisms are resistant to disinfectants at concentrations that reduce bacterial levels (*3*). Contamination of fresh produce could pose a health risk to humans because fresh produce is eaten raw. High levels of viral contamination can result in large outbreaks, but intermittent contamination of fresh produce accounts for some sporadic cases of norovirus and rotavirus gastroenteritis.

During April 27–November 23, 2009, we performed viral testing on 328 samples of packaged leafy greens (representing 12–14 different lots from 3–6 companies per week; no samples were taken on weeks with a statutory holiday) for norovirus or rotavirus RNA. Packaged leafy greens were purchased from retail stores in southern Ontario, Canada. Shipments maintained an average temperature of 3.8°C during transit to the testing laboratory. Each 25-g sample was spiked with 10^6^ PFU of feline calicivirus (FCV) as a sample process control (*4*). Virus was concentrated by using an adsorption-elution-ultrafiltration filtration protocol (*4*).

Recovery of FCV was quantified from an RNA standard curve. FCV process control recovery was <0.01% for 55 (17%) samples. Recovery of >0.01% of the FCV was observed for the remaining 273 (83%) samples. Two samples from which FCV was not recovered were positive for norovirus (CE-V-09–0138) and rotavirus (CE-V-09–0129); they were considered true positive results.

Of these 275 samples, 148 (54%) were positive for norovirus by real-time reverse transcription–PCR (RT-PCR) (*5*), and 1 (0.4%) was positive for rotavirus group A by RT-PCR (*6*). To confirm detection of norovirus RNA, we amplified a second norovirus target by RT-PCR of region C (*5*). Only 40 samples (15% of total) produced a band of the expected size for this second norovirus amplicon. Of these 40 amplicons, only 16 (6% of total) could be sequenced to confirm norovirus RNA. The rotavirus-positive sample was confirmed by sequencing.

For some sample dates, multiple lots were positive; for others, no positive samples were identified (Figure). Multiple detections on the same date were not caused by cross-contamination; partial capsid sequencing showed different genetic types on dates when multiple samples were positive (Figure). Results were positive from 5 different brands, and no organic samples were confirmed positive for enteric virus contamination. Of the 16 norovirus strains confirmed, 13 belonged to genogroup I (GI) and 3 to genogroup II (GII) (Figure). All were strain types known to be human pathogens. The group A rotavirus was not subtyped; group A rotaviruses can be human or animal pathogens.

**Figure:**
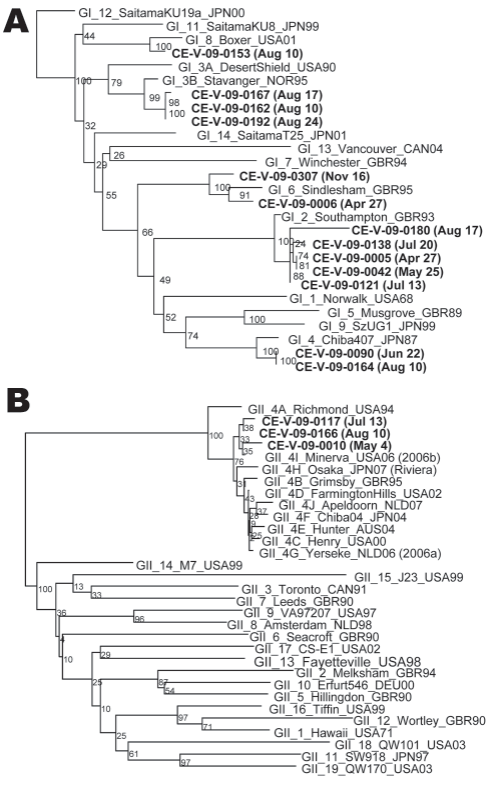
Phylogenetic analysis of the partial capsid sequence from genogroup I (A) and genogroup II (B) norovirus strains detected on leafy greens samples, Ontario, Canada, 2009, compared with the ViroNet Canada reference set for this region. Dates in parentheses are the date when testing was performed. Bootstrap scores were assigned as a percentage of 2,000 replicates.

Most noroviruses detected belonged to GI. Previous reports indicate that GI norovirus are more frequently identified in foodborne or waterborne outbreaks; GII.4 noroviruses are more common in large outbreaks spread person to person (*7*). Identification of GI norovirus is consistent with occasional contamination of produce or wash water. Disinfectants and sanitation agents are used in wash water at low concentrations, at which they have limited efficacy against norovirus (*3*).

Washing and disinfecting produce before eating it can reduce the risk for infection by reducing the viral load by 10- to 1,000-fold (*8*). The median level of confirmed contamination in this study was ≈500 RNA copies for norovirus (range 1.4 copies to 9 × 10^6^ copies).

A limitation of our findings is the inability to determine the association between molecular detection results and infectious virus. No outbreaks were related to the sequences detected here. There is no routine cell culture system for the laboratory growth of human norovirus. Genomic RNA can persist after the virus has been inactivated (*9*). The new ViroNet Canada network, which went online in April 2010, will monitor strains detected in leafy greens and other food products together with strains from community outbreaks to identify outbreaks linked to contaminated foods.

Our comprehensive surveillance study identified norovirus and rotavirus contamination of packaged leafy greens. We detected noroviruses on 6% and rotavirus on 0.4% of lots tested from retail markets in southern Ontario. Packages with confirmed positive samples were both imported into Canada and had been conventionally grown. Noroviruses have a low infectious dose (*2*), and detection of viral RNA is associated with human health risk in oysters, another commodity that is eaten raw (*10*). Our results suggest a possible risk for foodborne transmission of norovirus and rotavirus from packaged leafy greens.
