# Supplementary material for: Enteric Viruses in Ready-to-Eat Packaged Leafy Greens
Source: Emerg Infect Dis. 2010 Nov;16(11):1815–7. doi: 10.3201/eid1611.100877 (PMC3311121; doi:10.3201/eid1611.100877)
Supplement: Related texts

# Reply: Enteric Viruses in Ready-to-Eat Packaged Leafy Greens

**Authors:** Craig Hedberg

**Affiliations:** Author affiliation: University of Minnesota School of Public Health, Minneapolis Minnesota, USA

**In Response:** The report by Mattison et al. about detection of noroviruses in 6% of ready-to-eat packaged leafy greens sampled in Ontario, Canada, suggests that these products could be vehicles for widespread dissemination of norovirus (*1*). As they suggest, this finding should lead to studies evaluating the potential risk from such contamination. In particular, prospective attempts to identify whether these strains may be associated with community outbreaks are necessary. However, the primary norovirus genotype identified in the leafy greens samples (GI) is not the norovirus that has primarily caused human illness in recent years (GII).

Ready-to-eat packaged leafy greens are widely eaten. One third of respondents to the 2002 FoodNet Population Survey reported eating prepackaged salad in the week before interview (*2*). In the absence of evidence linking this contamination to norovirus outbreaks, it is premature for consumers to change how they handle or eat ready-to-eat packaged leafy greens.

The authors provide data on the apparent viral loads they observed and cite data to suggest that washing and disinfecting produce before eating it could reduce viral loads below the level of an infectious dose. However, the Food and Drug Administration does not recommend rewashing prewashed produce and does not recommend washing fresh produce with soap, detergent, or commercial produce washes (*3*). Because the products sampled in the study by Mattison et al. were prewashed, whether washing them would further reduce viral loads is not clear. In addition, rewashing ready-to-eat produce creates a potential risk for cross-contamination of the produce in consumers’ kitchens. Soap, detergents, or sanitizers could leave potentially harmful residues if rewashed produce is not thoroughly rinsed. These potential risks need to be weighed against the uncertain potential benefits of rewashing ready-to-eat packaged leafy greens. Given the ubiquity of these products, any change in recommended handling practices could have far-reaching consequences.

## References

[1] Mattison K, Harlow J, Morton V, Cook A, Pollari F, Bidawid S, Enteric viruses in ready-to-eat packaged leafy greens. Emerg Infect Dis. 2010;16:1815–7.2102956010.3201/eid1611.100877PMC3311121

[2] Centers for Disease Control and Prevention. Foodborne Diseases Active Surveillance Network (FoodNet): population survey atlas of exposures, 2002. Atlanta: The Centers; 2004. p. 1.

[3] Food and Drug Administration. Tips for fresh produce safety [cited 2010 Aug 16]. http://www.foodsafety.gov/keep/types/fruits/tipsfreshprodsafety.html
